# The clinical value of minimal invasive autopsy in COVID-19 patients

**DOI:** 10.1371/journal.pone.0242300

**Published:** 2020-11-11

**Authors:** Valentino D’Onofrio, Elena Donders, Marie-Elena Vanden Abeele, Jasperina Dubois, Reinoud Cartuyvels, Ruth Achten, Martin Lammens, Amelie Dendooven, Ann Driessen, Lukasz Augsburg, Jan Vanrusselt, Janneke Cox

**Affiliations:** 1 Faculty of Medicine and Life Sciences, Hasselt University, Hasselt, Belgium; 2 Department of Infectious Diseases and Immunity, Jessa Hospital, Hasselt, Belgium; 3 Department of Geriatrics, Jessa Hospital, Hasselt, Belgium; 4 Intensive Care and Anesthesiology, Jessa Hospital, Hasselt, Belgium; 5 Clinical Laboratory, Jessa Hospital, Hasselt, Belgium; 6 Department of Pathology, Jessa Hospital, Hasselt, Belgium; 7 Department of Pathology, Antwerp University Hospital, Edegem, Belgium; 8 Core, University of Antwerp, Wilrijk, Belgium; 9 Department of Pathology, University Hospital Ghent, Ghent, Belgium; 10 Department of Radiology, Jessa Hospital, Hasselt, Belgium; Ospedale Sant'Antonio, ITALY

## Abstract

**Background:**

Minimally invasive autopsy (MIA) is a validated and safe method to establish the cause of death (COD), mainly in low-resource settings. However, the additional clinical value of MIA in Coronavirus disease (COVID-19) patients in a high-resource setting is unknown. The objective was to assess if and how MIA changed clinical COD and contributing diagnoses in deceased COVID-19 patients.

**Methods and findings:**

A prospective observational cohort from April to May 2020 in a 981-bed teaching hospital in the epicenter of the COVID-19 pandemic in Belgium was established. Patients who died with either PCR-confirmed or radiologically confirmed COVID-19 infection were consecutively included. MIA consisted of whole-body CT and CT-guided Tru-Cut® biopsies. Diagnostic modalities were clinical chart review, radiology, microbiology, and histopathology which were assessed by two independent experts per modality. MIA COD and contributing diagnoses were established during a multi-disciplinary meeting. Clinical COD (CCOD) and contributing diagnosis were abstracted from the discharge letter. The main outcomes were alterations in CCOD and contributing diagnoses after MIA, and the contribution of each diagnostic modality. We included 18 patients, of which 7 after intensive care unit hospitalization. MIA led to an alteration in 15/18 (83%) patients. The CCOD was altered in 5/18 (28%) patients. MIA found a new COD (1/5), a more specific COD (1/5), a less certain COD (1/5), or a contributing diagnosis to be the COD (2/5). Contributing diagnoses were altered in 14/18 (78%) patients: 9 new diagnoses, 5 diagnoses dismissed, 3 made more specific, and 2 made less certain. Overall, histopathology contributed in 14/15 (93%) patients with alterations, radiology and microbiology each in 6/15 (40%), and clinical review in 3/15 (20%). Histopathology was deemed the most important modality in 10 patients, radiology in two patients, and microbiology in one patient.

**Conclusion:**

MIA, especially histological examination, can add valuable new clinical information regarding the cause of death in COVID-19 patients, even in a high-resource setting with wide access to premortem diagnostic modalities. MIA may provide important clinical insights and should be applied in the current ongoing pandemic.

**Trial registration:**

Clinicaltrials.gov identifier: NCT04366882

## Introduction

Minimally invasive autopsy (MIA) is a validated tool to establish the cause of death, that has been studied mainly in resource-limited settings [1]. One of the advantages of MIA is its limited risk of disease transmission making it an ideal tool in the current coronavirus disease 2019 (COVID-19) pandemic [2, 3]. However, if it increases clinical knowledge in COVID-19 patients in high-resource settings remains unknown. We systematically performed MIA in deceased COVID-19 patients and assessed to which extent clinically relevant diagnoses changed, compared to premortem diagnoses.

## Methods

This was a prospective observational cohort at Jessa hospital, Hasselt, Belgium. Patients with either severe acute respiratory syndrome coronavirus 2 (SARS-CoV-2) polymerase chain reaction (PCR) positivity or radiologically confirmed COVID-19 who died during hospitalization were consecutively included. Radiologically confirmed COVID-19 was defined as a person in whom PCR testing for COVID-19 is negative, but in whom the diagnosis is made on the basis of a suggestive clinical presentation AND a compatible CT-scan, according to the Belgian national guidelines. The researchers were notified by a mortuary staff member in case of new eligible patients. Autopsies of all included patients were performed maximally 24 hours after death. Relevant demographic data (age, sex, comorbidities, admission date and time of death) were collected from patient’s electronic medical file. Whole body 128-slice CT-scan was performed (Somatom go.top, Siemens Healthcare, Erlangen, Germany) followed by CT-guided Tru-Cut® biopsies. Four sterile lung biopsies were taken for microbiological examination and at least 2 biopsies from heart, lungs, liver, spleen, kidneys and abdominal fat for histological examination. Additional samples were taken when indicated. Each tissue was stained routinely with hematoxylin-eosin (H&E) and with ancillary staining when indicated. Lung tissue was inoculated on standard culture media for bacteria, yeasts and molds and microorganisms were identified by Matrix-assisted laser desorption ionization–time of flight mass spectrometry. SARS-CoV-2 real-time-PCR on lung tissue and IgG antibody detection was performed for all radiologically confirmed COVID-19 patients.

The clinical cause of death (CCOD) and contributing diagnoses were abstracted from the discharge letter by an independent researcher who was not part of the team of clinical reviewers. Two clinicians, two radiologists, two microbiologists and two pathologists independently assessed the clinical files, the postmortem CT-scans, microbiological findings and histology slides respectively. The clinical file review included the discharge letter. The other diagnostic modalities were assessed blinded from the CCOD.

During a multidisciplinary meeting the results of each diagnostic modality were presented, and the MIA cause of death (MCOD) and contributing diagnoses were formulated in consensus. Furthermore, the contribution of each diagnostic modality (clinical review, radiology, microbiology and histopathology) was assessed and ranked from most important to least important, or no contribution, during the meeting. Afterwards, the MCOD and contributing diagnoses were compared to the CCOD and contributing diagnoses. Alterations in CCOD and contributing diagnoses were specified. Descriptive statistics were used to report the proportion of diagnoses that were altered by MIA, how they were altered and how the different modalities contributed.

Patients were included after oral informed consent was obtained from their legal representative. Oral consent was documented together with patient and legal representative contact information in a data file stored on a secured server in the hospital. Written consent of the legal representative could not be obtained due to visiting restrictions in the hospital during the pandemic. An information sheet containing the contact details of the researcher was send by registered mail. The study and the procedure for oral consent received ethical approval from the Ethics Committee of Jessa hospital and Hasselt University (Clinicaltrials.gov identifier: NCT 04366882).

## Results

We included 18 out of 25 eligible patients (72%) between 14^th^ of April and 12^th^ of May, of which 15 were PCR SARS-CoV-2 and 3 radiologically confirmed. For the excluded patients, consent was declined by the legal representative (n = 4) or the legal representative could not be reached (n = 3). The median (interquartile range [IQR]) age was 80 years (72–84), 10/18 (56%) patients were male, median (IQR) Charlson Comorbidity index was 3 (1–4) and 12/18 (67%) patients had a no invasive-ventilation policy. In total 7/18 (39%) patients were admitted to the ICU at time of death and the median (IQR) time from admission to death was 18 days (5–22). All but 2 patients had respiratory failure (need for invasive ventilation or PaO_2_/FiO_2_ ratio <300) in the 24 hours preceding death. In the 72 hours before death, 9/18 (50%) patients received broad-spectrum antibiotics or antifungals and 13/18 (72%) anticoagulants.

In 15/18 (83%) patients, MIA led to an alteration in CCOD or contributing diagnosis: in 5/18 patients the CCOD altered and in 14/18 a contributing diagnosis was changed (Table 1). MIA revealed the COD in one patient, i.e. radiological COVID-19 with severe pneumonia as CCOD was dismissed and heart failure revealed as MCOD. CCOD was made more specific in one patient and less certain in another. In two patients, conditions that were determined clinically as contributing diagnoses were deemed more relevant by MIA and assigned as the MCOD.

**Table 1:** Premortem clinical cause of death and contributing diagnoses and postmortem MIA cause of death and contributing diagnosis per patient.

Patient	Disease duration LOS (days)	ICU admission Invasive ventilation		Clinical COD Clinical contributing diagnoses	MIA COD MIA contributing diagnoses	MIA alteration
**PCR confirmed COVID-19 patients**						
1	21	Yes	COD	Rabdomyolysis with subsequent MOF including renal failure with dialysis	Rabdomyolysis eci with subsequent MOF including renal failure with dialysis	Confirm
	20	Yes	Contributing diagnoses	COVID-19 severe pneumonia clinically improving	COVID-19 severe pneumonia	Confirm
2	51	Yes	COD	Sudden death eci	Sudden death eci	Confirm
	44	Yes	Contributing diagnoses	COVID-19 severe pneumonia clinically improving	COVID-19 severe pneumonia clinically improving	Confirm
					Minor intracerebral bleeding	New
					Sepsis	New
3	41	No	COD	Acute on chronic renal failure	Acute on chronic renal failure due to crescentic glomerulonephritis	More specific
	23	No	Contributing diagnoses	COVID-19 infection, clinical uncertainty if pneumonia	COVID-19 severe pneumonia	More specific
				Bacterial co-infection highly suspected		Dismiss
4	8	No	COD	COVID-19 severe pneumonia	Massive pulmonary embolism	Confirm (Assign as immediate COD)
	7	No	Contributing diagnoses	Massive pulmonary embolism	COVID-19 severe pneumonia	Confirm
				Hepatitis eci	Right sided heart failure leading to severe sinusoidal dilatation in the liver	More specific
5	Unkown	Yes	COD	Intracerebral bleeding	Intracerebral bleeding	Confirm
	20	Yes	Contributing diagnoses	Renal failure eci leading to dialysis	Renal failure due to ATN leading to dialysis	More specific
				COVID-19 severe pneumonia	COVID-19 severe pneumonia	Confirm
					Steatohepatitis	New
6	27	No	COD	COVID-19 severe pneumonia	COVID-19 severe pneumonia	Confirm
	17	No	Contributing diagnoses	Acute on chronic renal failure	*No renal biopsy performed*	-
					Left-and right sided heart failure	New
					Subileus	New
7	18	No	COD	Probable invasive *Aspergillus fumigatus* pulmonary infection	COVID-19 severe pneumonia	Confirm (Assign as immediate COD)
	17	No	Contributing diagnoses	COVID-19 severe pneumonia	Probable invasive *Aspergillus fumigatus* pulmonary infection	Less certain
				Cerebral B-cell lymphoma	Cerebral B-cell lymphoma	Confirm
8	Unkown	No	COD	Small cell lung carcinoma with metastasis	Small cell lung carcinoma with metastasis	Confrim
	4	No	Contributing diagnoses	COVID-19—mild illness	COVID-19—mild illness	Confirm
					Pancreatitis eci	New
9	3	No	COD	COVID-19 severe pneumonia	COVID-19 severe pneumonia	Confirm
	1	No	Contributing diagnoses	Bacterial COPD excacerbation		Dismiss
10	18	Yes	COD	Intracranial bleeding with subdural hematoma	Intracranial bleeding with subdural hematoma	Confirm
	3	No	Contributing diagnoses	COVID-19 severe pneumonia	COVID-19 severe pneumonia	Confirm
11	32	Yes	COD	Para-tracheal bleeding eci while on anticoagulant therapy for DVT and AF	Para-tracheal bleeding eci while on anticoagulant therapy for DVT and AF	Confirm
	26	No	Contributing diagnoses	COVID-19 severe pneumonia	COVID-19 severe pneumonia	Confirm
12	28	Yes	COD	COVID-19 severe pneumonia	COVID-19 severe pneumonia	Confirm
	24	No	Contributing diagnoses	Hospital acquired pneumonia	Hospital acquired pneumonia	Less certain
13	Unkown	No	COD	Hemorrhagic and semi recent ischemic cerebrovascular accident	Hemorrhagic and semi recent ischemic cerebrovascular accident	Confirm
	19	No	Contributing diagnoses	Depression with refusal of food and medical interventions	Depression with refusal of food and medical interventions	Confirm
				COVID-19—mild illness	COVID-19—mild illness	Confirm
					Bacterial pneumonia	New
14	Unkown	Yes	COD	COVID-19 pneumonia	COVID-19 pneumonia	Confirm
	22	No	Contributing diagnoses	Hospital acquired pneumonia		Dismiss
					Left- and right sides heart failure	New
15	12	No	COD	COVID-19 Pneumonia	COVID-19 Pneumonia	Confirm
	65	No	Contributing diagnoses	Post-anoxic encephalopathy after out-of-hospital cardiac arrest	Post-anoxic encephalopathy after out-of-hospital cardiac arrest	Confirm
				Hospital acquired pneumonia		Dismiss
**Radiologivally confirmed COVID-19 patients**						
16	Unkown	No	COD	Radiological COVID-19 severe pneumonia with negative SARS-CoV-2 PCR	Left-and right sided heart failure	Dismiss/New
	6	No	Contributing diagnoses	Pseudoaneurysma left femoral artery	Pseudoaneurysma left femoral artery	Confirm
17	1	No	COD	Radiological COVID-19 severe pneumonia with negative SARS CoV-2 PCR	Viral pneumonia	Less certain
	1	No	Contributing diagnoses		Left- and right sided heart failure	New
18	11	No	COD	Bacterial Pneumonia	Bacterial pneumonia	Confirm
	1	No	Contributing diagnoses	Left- and right sided heart failure	Left- and right sided heart failure	Confirm
				Radiological COVID-19 severe pneumonia with negative SARS CoV-2 PCR considered		Dismiss

Per patient, the COD is the first diagnosis given. Following diagnoses are contributing findings. eci: e causa ignota; MOF: multi-organ failure; COVID-19: corona viral disease 2019; ATN: acute tubules necrosis; SARS-CoV-2: severe acute respiratory syndrome coronavirus 2; PCR: polymerase chain reaction; COPD: chronic obstructive pulmonary diseases; DVT: deep venous thrombosis; AF: atrial fibrillation

MIA revealed 9 new contributing diagnoses, 5 contributing diagnoses were dismissed after MIA, 3 made more specific, and 2 made less certain.

For all 3 patients with radiologically confirmed COVID-19 both postmortem serology and PCR on lung tissue were negative. Two of these patients died of bacterial pneumonia and heart failure respectively, and the diagnosis COVID-pneumonia was completely dismissed. For the third patient, MIA concluded an unspecified viral pneumonia as MCOD. When considering only PCR confirmed COVID-19 patients, MIA led to an alteration in 12/15 (80%) patients: in 3/15 patients the CCOD was altered and in 12/15 a contributing diagnosis was changed.

Bacterial pneumonia as clinical contributing diagnosis was dismissed in 3 patients and made less certain in one. Overall, MIA found bacterial or fungal pneumonia as relevant diagnosis in only 2/18 (11%) patients.

When assessing the 15 patients in which MIA contributed to the final diagnoses, histopathology contributed in 14/15 (93%) patients, radiology and microbiology each in 6/15 (40%) patients, and clinical review in 3/15 (20%) patients. When ranked according to contribution, histopathology ranked first in 10 patients, and second and third in one patient each. Radiology was ranked first in 2 patients, and microbiology in 1 patient. In 2 patients, each modality contributed equally.

## Discussion

MIA led to alterations in CCOD and contributing clinical diagnoses in 83% of deceased patients with either PCR-confirmed (15 patients) or radiologically confirmed (3 patients) COVID-19. Ten clinically relevant diagnoses were revealed. These included heart failure (four times), sepsis, and bacterial pneumonia, i.e. diagnoses that might have influenced clinical treatment when known premortem. In all patients with radiologically confirmed COVID-19, SARS-CoV-2 infection could not be confirmed with postmortem PCR or serology.

In 2/3 radiologically confirmed COVID-19 patients, MIA dismissed the diagnosis of COVID-19 altogether, and in one, it made COVID-19 very unlikely. These were patients that were isolated and treated as COVID-19 patients, in line with the Belgian national guidelines [4]. International guidelines also include patients with typical chest findings as probable COVID-19 cases, as PCR testing for SARS-CoV-2 does not have 100% sensitivity [5, 6]. Although we included only 3 radiological COVID-19 patients, our results confirm the lack of specificity for COVID-19 on CT-scans [7] and emphasize the need for clinicians to remain alert in these cases, even amid a pandemic, and consider alternative diagnoses [7].

Overall, MIA found histopathological or microbiological evidence of bacterial or fungal superinfection in 11% of patients, yet 50% of patients were on antibiotic and/or antifungal treatment in the 72 hours before death. Even though MIA results could have been negatively influenced by concurrent antimicrobial treatment or sampling error (although sampling was done by CT-guidance), this observation is in line with others reporting low prevalence of co-infections [8–11]. This is of relevance as antimicrobial overuse leads to resistance, toxicity and unnecessary costs.

Histopathology was the diagnostic modality within MIA that most often contributed to the final conclusion, and therefore considered the most relevant part of MIA. Radiology was found to have less impact. This may be partly explained by the fact that 6/18 (33%) of patients had a CT-scan 48 hours prior to death, showing relevant findings in all six. Therefore, if CT-scanning was not as widely available premortem, it would have had a higher postmortem yield.

Inherent to its technique, MIA may not be able to detect all clinically relevant findings. For example, for pulmonary embolism—an important complication in COVID-19 patients [12, 13]—MIA has insufficient sensitivity. Therefore, complete autopsies remain the gold standard to establish the COD. However, complete autopsy rates have been decreasing in high-income setting over the last decades [14], with likely simultaneous loss of expertise and facilities to perform complete autopsies. Moreover, acceptance of MIA by relatives may be higher when compared to complete autopsy [1]. Lastly, in the beginning of the COVID-19 pandemic, there was uncertainty about the safety of performing complete autopsies and reluctance to perform them [3, 15, 16]. Therefore, we think MIA should be viewed as an additional method to gain clinically relevant insights, especially when complete autopsies are not feasible.

One of the strengths of this study is the prospective and consecutive inclusion of patients for autopsy, and thus the absence of selection based on disease severity. On the other hand, this study has some limitations. First, sampling was limited to certain organs, e.g. we found in 33% of our patients relevant radiological abnormalities in the brain but because the brain was not biopsied, a more precise diagnosis could not be made. Second, some patients had treatment restrictions during admission, limiting diagnostic and therapeutic management during life, which may have biased our findings. Furthermore, discharge letters may not provide the complete clinical picture premortem, although the postmortem clinical file review only contributed to the MIA final diagnosis in 3/18 patients. Lastly, the distinction between COD and contributing diagnosis is often artificial. Patients die as a result of a cascade of events, influenced by numerous external factors. A list of diagnoses cannot simply reflect the disease complexity [17].

Our study shows that MIA adds clinically relevant information on COD and contributing diagnoses in COVID-19 patients in a majority of patients, also in a high-technological setting. More accurate diagnoses provide a better knowledge on what diseases eventually cause death in COVID-19 patients and informs and improves future care. For that purpose, MIA can be applied in the current ongoing pandemic.
